# Transcranial Magnetic Stimulation in Pregnancy: Efficacy, Safety, and Future Implications for Perinatal Mental Health Care

**DOI:** 10.1002/brb3.70304

**Published:** 2025-02-09

**Authors:** Serena Angeline, Babangida Tiyatiye, Wole Akosile

**Affiliations:** ^1^ Prince Charles Hospital Metro North Mental Health Brisbane Queensland Australia; ^2^ Perth Clinic, School of Medicine The University of Western Australia Perth Western Australia Australia; ^3^ New Farm Clinic, Faculty of Health and Behavioural Sciences, National Centre for Youth Substance Use Research University of Queensland Brisbane Queensland Australia

**Keywords:** depression, obstetric, pregnancy, rTMS, TMS, transcranial magnetic stimulation

## Abstract

**Introduction:**

Repetitive transcranial magnetic stimulation (TMS) has gained interest as a treatment for major depressive disorder (MDD). However, the literature on its efficacy and safety for pregnant patients with MDD is limited. This article reviews and appraises available studies on TMS in pregnant women with MDD.

**Methods:**

We reviewed randomized controlled trials and open‐label studies on TMS in pregnant women with MDD.

**Results:**

Studies indicate that TMS is a safe and effective treatment for MDD during pregnancy, showing significant reductions in depression scores and increased response and remission rates compared to sham TMS. TMS was well tolerated with minimal side effects.

**Conclusion:**

Larger, multicenter trials are needed to develop evidence‐based protocols for TMS use in pregnancy.

## Graphical abstract

TMS for Depression in Pregnancy:

Safe & Effective Alternative.
Significant reduction in depression scores.
Increased response & remission rates vs. sham.
Well‐tolerated with minimal or no side effects.
Promising non‐pharmacological option.
Further large‐scale studies are needed.
It might be a safe and viable option for rapid treatment of depression in Mother‐baby units.

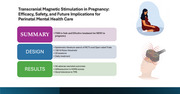

## 1 Introduction

Major depressive disorder (MDD) is estimated to affect between 7% and 12% of pregnant women worldwide (Bennett et al. 2004) and is a major contributor to disease burden in both developed and developing countries across the globe (Adewuyi et al. 2023; Akosile et al. 2023; Dadi et al. 2020) Untreated depression during pregnancy has been associated with a myriad of adverse outcomes, notably encompassing an elevated propensity for premature delivery, gestational hypertension, low birth weight, perinatal mortality, detrimental effects on the quality of infant‐maternal bonding, and enduring repercussions on infant emotional and cognitive development (Jahan et al. 2021; Jarde et al. 2016; Malhi et al. 2021). Consequently, it is paramount to emphasize the significance of assertive therapeutic interventions to optimize the overall well‐being of the expectant mother and her neonate (Jahan et al. 2021; Malhi et al. 2021). To date, guidelines for the mainstay of treatment of depression in pregnancy have predominantly consisted of psychotherapy in mild‐moderate depression, pharmacotherapy, or electroconvulsive therapy (ECT) in severe cases (Malhi et al. 2021; National Collaborating Centre for Mental Health, 2014). Nonetheless, it is imperative to acknowledge that pharmacotherapy, while a viable approach for treating depression during pregnancy, is accompanied by a constellation of notable side effects (Beck‐Pancer et al. 2023; MacDonald, Wimalaguna, and Akosile 2019; Martin et al. 2023; Wang, Ying, and Jiang 2023). These side effects encompass but are not limited to, a reduction in birth weight, the potential for congenital malformations, the risk of persistent pulmonary hypertension, susceptibility to postpartum hemorrhaging, and the emergence of withdrawal symptoms in the neonate (Anderson et al. 2020). ECT as an alternative therapeutic modality is not without its own set of well‐documented risks (Arnison et al. 2023). These documented risks encompass concerns about the administration of anesthesia, the occurrence of induced seizures, cognitive deficits in the form of memory loss, as well as the potential for inducing premature contractions and preterm labor in pregnant individuals (Anderson and Reti 2009; Arnison et al. 2023; Leiknes et al. 2015). Furthermore, stigma associated with ECT, unfortunately, continues to be a significant barrier in the treatment of individuals with major depressive disorder (Goldbloom and Gratzer 2020). More recently, transcranial magnetic stimulation (TMS) has been postulated as a treatment for depression (Akosile et al. 2023; Sonmez et al. 2019).

TMS, first developed by Barker and colleagues in 1985, was initially introduced to the field of medicine for research purposes (Dallas Hamlin and John Garman 2023). Based on Faraday's principles of electricity and magnetism, it was studied by researchers over the next few decades to gain insights into its potential applications in clinical medicine, specifically psychiatry and neurology, through the understanding of its potential for neuromodulation of the prefrontal cortex (Peng et al. 2018). After extensive research investigating its mechanism of action, efficacy and safety, in the early 2000s, TMS began gaining increasing traction with its use in the treatment of psychiatric disorders, with a specific focus on major depressive disorder. Gaining FDA approval in 2018, TMS rapidly gained global interest in its potential to treat a range of psychiatric and neurological conditions (Dallas Hamlin and John Garman 2023).

Since its conception in 1985, an introduction to clinical applications in the 2000s, TMS has shown encouraging results in the treatment of depression and is typically associated with fewer adverse effects in comparison to antidepressant medications as well as to ECT (Chen et al. 2017; Li et al. 2021; Miuli et al. 2023). Considering the significant side effects associated with antidepressant medications during pregnancy, the consideration of alternative treatments becomes paramount. Transcranial magnetic stimulation (TMS) emerges as a noteworthy option, offering a nonpharmacological approach to managing depression in pregnant individuals.

Antidepressants, particularly selective serotonin reuptake inhibitors (SSRIs), are commonly prescribed during pregnancy (Ailes et al. 2016; Sun et al. 2019). However, the potential risks, including a low but notable risk of specific birth defects in the first trimester, pose concerns that necessitate careful evaluation. The increased risk of preterm birth and the potential for neonatal withdrawal syndrome further underscore the complexities associated with antidepressant use during pregnancy. These considerations highlight the urgency of exploring alternative interventions that minimize potential harm to the expectant mother and the developing fetus.

TMS presents itself as a promising option in this context. As a noninvasive and nonpharmacological treatment, TMS avoids the potential side effects associated with antidepressants. Its application involves using magnetic fields to stimulate specific areas of the brain implicated in depression, offering a targeted and precise therapeutic approach. Importantly, TMS does not involve the introduction of chemicals into the body, thus mitigating concerns related to drug exposure during pregnancy. However, safety concerns regarding its use in pregnant women, including the risk of seizure provocation, remains the most serious potential adverse event associated with TMS (Rossi et al. 2021). Although the risk is very low, especially with traditional stimulation parameters and focal coils, it warrants careful consideration in pregnant patients. Another crucial aspect is the magnitude of the induced electric field generated by TMS and its potential interaction with the developing fetus. While current evidence suggests that the electric field generated is far below the safety limit recommended for pregnant patients, the long‐term effects of fetal exposure to these fields remain uncertain and require further investigation (Rossi et al. 2021).

While recognizing the imperative to address maternal depression for the well‐being of both the mother and the unborn child, the potential risks associated with antidepressant medications underscore the importance of considering alternative modalities. TMS, with its favorable safety profile, provides a viable and potentially advantageous option for managing depression during pregnancy. As with any treatment decision, open discussion and collaboration between the pregnant individual and their healthcare provider are crucial to tailor an approach that prioritizes both the mental health of the mother and the safety of the developing fetus. This underscores the need for ongoing research and awareness regarding TMS as a valuable therapeutic option in the context of pregnancy‐related depression.

It has been included in many international treatment guidelines as an effective modality in the management of treatment‐resistant depression (Malhi et al. 2021). Over the past decade, there has been a notable uptick in various reports highlighting the successful treatment of depression in pregnant women using TMS. These reports have included numerous case reports, open‐label trials, and randomized controlled trials. The primary objective of this paper is to engage in a qualitative assessment of the extant literature, focusing specifically on the safety and efficacy of TMS as a therapeutic intervention for depression during pregnancy.

## 2 Materials and Methods

Data Sources and Search Strategy: A systematic literature search was conducted by comprehensively querying the following databases: EMBASE, MedLINE, PsychINFO, and CINAHL. The search employed the following keywords and their variations: “TMS,” “transcranial magnetic stimulation,” “rTMS,” in conjunction with “pregnancy,” “pregnant,” “fetal,” or “foetal.”

### 2.1 Study Selection Criteria

Types of Studies: This review encompassed Randomized Controlled Trials (RCTs) and open‐label trials, focusing on TMS as a treatment intervention for depression during pregnancy.

### 2.2 Inclusion Criteria

Studies that centered on pregnant women in any trimester of pregnancy.

Studies that specifically targeted individuals diagnosed with unipolar major depressive disorder.

**FIGURE 1:**
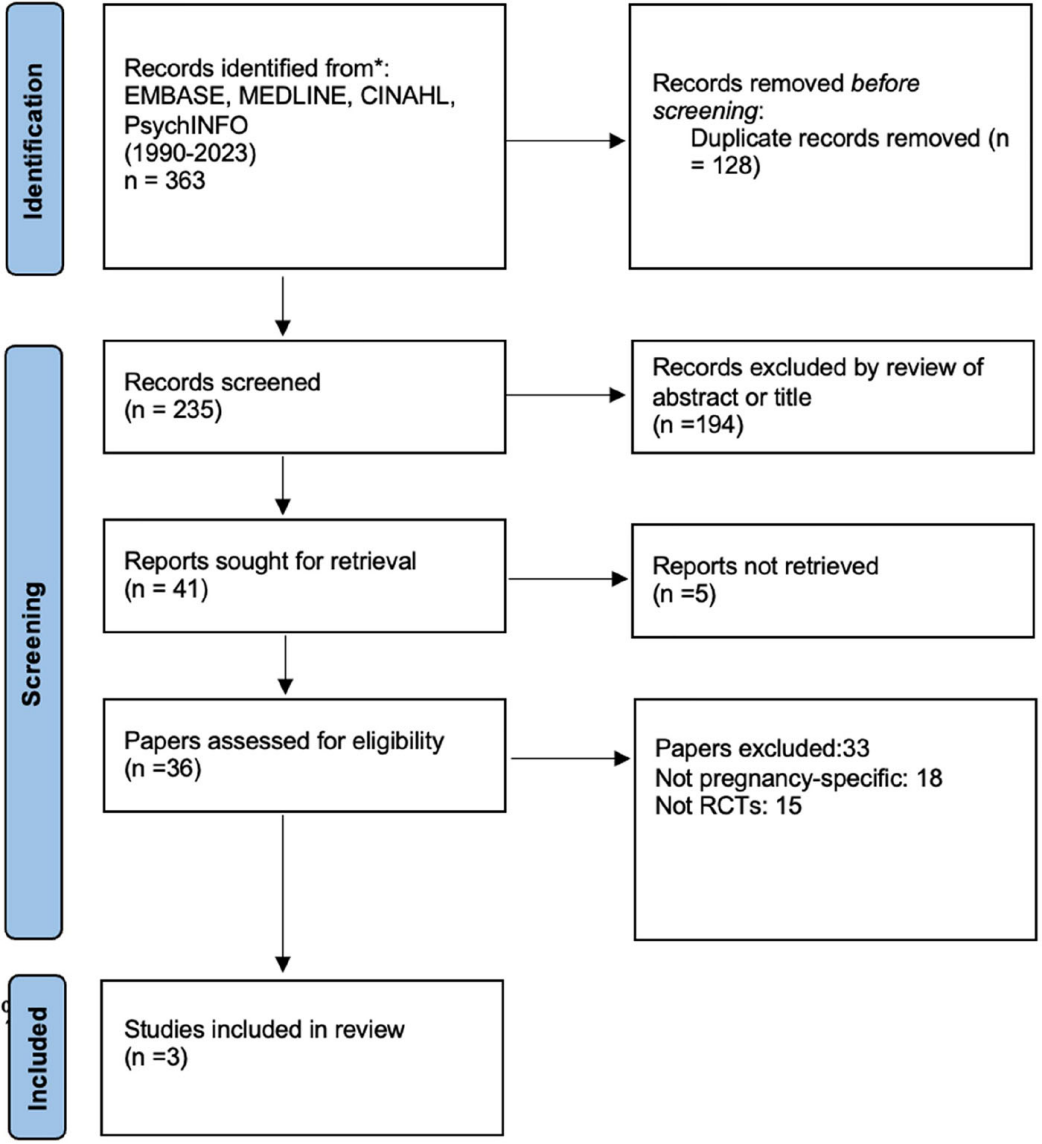
Flow chart.

No restrictions were imposed on concurrent or prior pharmacotherapy usage before or during TMS treatment (see Figure 1).

### 2.3 Exclusion Criteria

Studies concentrated on the peripartum period rather than the antenatal phase.

Studies utilizing TMS for indications unrelated to unipolar major depressive disorder (e.g., bipolar disorder, migraines, akathisia).

Studies employ TMS for maintenance purposes during pregnancy, as opposed to the acute treatment of a major depressive episode.

Studies not published in the English language were also excluded from consideration.

The study selection process was executed in a stepwise manner, with two independent reviewers screening titles and abstracts for initial eligibility. Subsequently, the full texts of potentially relevant articles were evaluated for final inclusion in the review. Any discrepancies were resolved through consensus or consultation with a third reviewer, as necessary. The following information was extracted from each article motor threshold: site of stimulation, frequency, number of pulses, interevent interval, number of sessions pre‐TMS depression rating, post‐TMS depression rating, response rate, response rate definition and neonatal outcomes (Table 1).

**TABLE 1:** Summary of studies included in review.

Study	**Kim et al**. (2011)—open‐label pilot (*N* = 10)	Kim et al. (2019)—RCT (*N* = 22)	Hizli Sayar et al. (2014)—open‐label 2014 (*N* = 30)
Motor threshold	100% MT	100% MT	100% MT
Site of stimulation	Right DLPFC	Right DLPFC	Left PFC
Frequency	1 Hz	1 Hz	25 Hz
Number of pulses	300	900 per session (18,000 total)	1000 per session
Interevent interval	Daily	Once daily on weekdays for 4 weeks	Daily, 6 days a week for 3 weeks
Number of sessions	20 sessions	20 sessions	18 sessions
Pre‐TMS depression rating	23.2 ± 3.5	23.18 ± 3.54 on HDRS	26.77 ± 5.58 on HAMD
Post‐TMS depression rating	9.3 ± 6.0	9.27 (±6.05)	13.03 ± 6.93
Response rate	70%	81.8% (A), 45.45% (S)	41.4% responded. 20.7% achieved remission
Response rate definition	≥ 50% decrease in HDRS	50% decrease from initial HDRS‐17 score	50% reduction in HAMD score
Neonatal outcomes	Nil adverse. All healthy at delivery, all >37 weeks, all APGARs healthy, no congenital issues	Late preterm birth in 3 out of 22 deliveries. All other maternal and delivery outcomes normal.	23 women gave birth to 25 healthy babies. 6 babies still in intensive OPD follow‐up, but no sign of IUGR

The PRISMA guidelines were adhered to in order to ensure the methodological rigor of this systematic review.

## 3 Results

Our systematic search yielded 235 articles, which were screened and relevant articles sorted for retrieval. Following a meticulous screening process and rigorous evaluation of these articles, only three studies met the stringent inclusion criteria, as depicted in the PRISMA diagram below. These included two open‐label studies and one Randomized Controlled Trial (RCT) (see Table 1).

It is noteworthy that the search results encompassed a multitude of case reports and case series, which were deliberately omitted from the final selection. The authors made the decision to exclude these case‐based studies collectively to maintain the focus and robustness of this review. The selected studies met the predefined methodological criteria and thus were deemed the most pertinent sources for our investigation.

The first study was a small open‐label pilot study conducted by Kim et al. (2011). This investigation enrolled a cohort comprising ten pregnant women in the advanced stages of their second or third trimester of pregnancy. Each of the participants bore the diagnosis of major depressive disorder according to the Diagnostic and Statistical Manual of Mental Disorders, Fourth Edition (DSM‐IV) criteria. Inclusion in the study was contingent upon the presence of a current depressive episode characterized by at least a moderate level of severity. Severity of the ongoing depressive episode was assessed through two validated measures: the Clinical Global Impression Severity Scale (with a score of ≥ 4 as the threshold) and the Hamilton Depression Rating Scale (with a score of ≥ 14 as the criterion for inclusion) (Busner and Targum 2007; Carrozzino et al. 2020).

Importantly, all subjects maintained a consistent and stable dosage of antidepressant medication prior to the commencement of transcranial magnetic stimulation (TMS) therapy, and there were no alterations to their antidepressant regimens throughout the course of the study. The TMS intervention employed in this study entailed the administration of 20 sessions. Each session consisted of transcranial magnetic stimulation delivered at a frequency of 1 Hz, with the magnetic intensity calibrated at 100% of the motor threshold. The target site for TMS application was the right dorsolateral prefrontal cortex. It is imperative to highlight that this study adopted an open‐label design, thus precluding the implementation of a blinding process. This transparency was a deliberate methodological choice, enabling the subjects to be aware of the therapeutic intervention they were receiving. TMS was delivered on a daily basis during weekdays over a span of 4 weeks, and a significant amelioration in the severity of depressive symptoms was observed. Notably, 70% of the patients exhibited a positive response to the treatment, which is defined as a reduction of at least 50% in their Hamilton Depression Rating Scale (HDRS) scores. Encouragingly, within this group, 30% met the criteria for remission, signifying a post‐TMS HDRS score of less than 8. Furthermore, the mean HDRS scores demonstrated a substantial reduction from an average of 24.4 prior to TMS to 9.7 following TMS intervention (*p* = 0.005). All ten women who participated in the study delivered healthy infants at full term, and no adverse neonatal outcomes were reported. This noteworthy outcome underscores the safety of the TMS treatment approach employed in this investigation with regard to neonatal health and well‐being.

This study was followed by a randomized controlled trial by the same authors in 2019 (Kim et al. 2019). This RCT encompassed 22 pregnant women who were diagnosed with major depressive disorder during their second or third trimester. These participants were randomly assigned to receive either active transcranial magnetic stimulation (TMS) or sham TMS. Each group underwent a course of 20 TMS sessions, where TMS was administered at a frequency of 1 Hz to the right dorsolateral prefrontal cortex, calibrated at 100% of the motor threshold. In addition to evaluating pre‐ and postintervention depression ratings, the study also measured estradiol and progesterone levels before the initial TMS session and after the twentieth session. Two participants withdrew prematurely from the study due to time commitment constraints.

The results revealed a substantial reduction in the HDRS score, with the mean HDRS score decreasing from 23.18 prior to TMS to 9.27 following the twentieth session. A noteworthy 81.8% of active TMS participants achieved the predefined criterion for response, characterized by a 50% reduction in HDRS scores, in contrast to 45.5% of sham TMS participants. Moreover, remission, defined as achieving HDRS scores lower than 8, was observed in 27.3% of the active TMS group and 18.8% of the sham TMS group. However, it is pertinent to note that neither the remission nor response rates achieved statistical significance, which the authors attributed to the limited sample size.

This RCT was the first to study pre‐ and postintervention estradiol or progesterone levels in pregnant women receiving TMS, noting that this was also not an outcome the authors studied in their previous 2011 open‐label trial. This RCT did not identify significant differences in pre‐ and postintervention estradiol or progesterone levels. Concerning neonatal outcomes, three out of the 22 participants experienced pre‐term births, with gestational ages of 35.3, 36.3, and 35.2 weeks. One infant in the active TMS group presented an abnormal initial assessment, possibly due to shoulder dystocia, unrelated to TMS. However, all other neonatal outcomes, including APGAR scores, birth length, and weight, fell within the normal range. Furthermore, the authors noted that only five women in the study were concurrently using psychiatric medication, and all of them were on stable doses for at least 2 weeks prior to commencing their TMS treatment.

In 2013, Hizli Sayer et al. conducted an open‐label study in which 30 pregnant women with major depressive disorder as diagnosed by DSM IV criteria, were treated with 20 sessions of 25 Hz TMS to the left prefrontal cortex (Hızlı Sayar et al. 2014). In this study, patients received a total of 18 TMS sessions, with each session administered daily, 6 days a week, spanning over a duration of 3 weeks. The mean gestational age at the first TMS session was 14.26 ± 8.25 weeks. The efficacy of the TMS treatment was assessed by measuring HDRS scores before the first session and after the twentieth session. The results indicated a significant reduction in mean HDRS scores, which decreased from 26.77 to 13.03. Importantly, 41.4% of the patients exhibited a favorable response to the TMS intervention, defined as a reduction of 50% or more in HDRS scores, while 20.7% achieved remission. Notably, there were no reports of adverse neonatal outcomes in association with this TMS treatment. This underscores the safety of the intervention concerning neonatal health. This study did not provide information or commentary on the concurrent use of psychiatric medication, including details regarding dosages and dose adjustments during or in proximity to the TMS treatment.

In all three studies, subjects exhibited good tolerance to TMS. The most frequently reported side effect across the three studies was a mild headache. The randomized controlled trial conducted by Kim et al. also noted infrequent, transient side effects, such as dizziness, nausea, site pain, supine hypotension, jaw discomfort, and eye twitch. However, it is noteworthy that these side effects did not exhibit statistical significance when comparing different treatment groups. Two studies performed low‐frequency TMS, which is associated with a lower risk of seizures compared to high‐frequency TMS (Kim and Paik 2021). However, none of the studies had seizures as an adverse event. Seizure risk in TMS is rare (Kim and Paik 2021).

### 3.1 Risk of Bias Assessment

The risk of bias for each study was evaluated employing the JADDAD tool for randomized controlled trials, and ROBINS tool for open‐label trials.

### 3.2 Open‐Label Studies

Both the 2013 study by Kim et al. and the study conducted by Hazli Sayar were classified as being at a serious risk of bias. This designation was primarily attributable to their open‐label nature, which inherently introduced a greater potential for bias. Factors contributing to this risk included the absence of blinding, an inadequately addressed assessment of confounding variables, and the potential for selection bias.

### 3.3 Randomized Controlled Trials (RCTs)

In contrast, the RCT conducted by Kim et al. received a low risk of bias rating according to the JADDAD tool. This favorable assessment was likely due to the structured and controlled design inherent to randomized trials, which typically include blinding procedures and rigorous measures to mitigate potential biases.

## 4 Discussion

The findings from the studies included in this review collectively suggest that TMS represents a potentially safe and effective therapeutic avenue for managing major depressive disorder during pregnancy. The studies included in this review consistently reported a significant reduction in mean HDRS scores in participants treated with TMS, with minimal or negligible adverse maternal and fetal effects. Each of these studies concluded that TMS was safe and effective and held significant promise in the treatment of pregnant individuals with major depressive disorder. Nonetheless, it is crucial to recognize the inherent limitations of this review. Chief among these limitations is the sparse literature dedicated to the subject, with a substantial portion of the available studies consisting of small sample size and bearing moderate to serious risk of bias assessments.

A fundamental constraint pertains to the paucity of studies examining the efficacy and safety of TMS during pregnancy. To date, only one randomized controlled trial (RCT) has been conducted in this domain, and this RCT was characterized by a relatively modest sample size of 22 participants. The authors commented that this study was underpowered, and based on their initial data calculations, the number of women required to complete an adequately powered trial would have been 33 women per arm—thus conveying that this study was able to recruit only 33% of the ideal minimum sample size. Moreover, the open‐label trials, which also had limited sample sizes, were similarly marred by a designation of serious bias risk. Consequently, while the findings from these studies provide encouraging insights into the potential utility of TMS for managing major depressive disorder in pregnancy, it is prudent to underscore the pressing need for larger‐scale and more robust RCTs to yield conclusive results.

A limitation arising from small sample sizes is the amalgamation of participants with Major Depressive Disorder (MDD) and treatment‐resistant MDD in collective analyses rather than conducting separate subgroup analyses. Widely acknowledged mood disorder guidelines, such as the Australian RANZCP guidelines (Malhi et al. 2021), emphasize transcranial magnetic stimulation's (TMS) primary utility in managing treatment‐resistant depression, defined as “an inadequate response to at least two adequate (appropriate dose and lasting for at least 6 weeks) treatment episodes with different drugs” (Voineskos, Daskalakis, and Blumberger 2020).

Challenges encountered during recruitment resulted in a participant cohort comprising a heterogeneous mix of individuals with MDD and treatment‐resistant MDD. Uncertainties persist regarding the extent of prior treatment attempts, introducing variability, and a lack of clarity on concurrent treatment modalities, particularly concomitant pharmacotherapy use. For example, in Kim et al.’s randomized controlled trial (RCT), 5 out of 22 female participants were on stable doses of pharmacotherapy for at least 2 weeks before the study, ranging from Selective Serotonin Reuptake Inhibitors (SSRIs) and Serotonin‐Norepinephrine Reuptake Inhibitors (SNRIs) to mood stabilizers and Norepinephrine‐Dopamine Reuptake Inhibitors (NDRI).

Future research would benefit from a more rigorous approach to participant inclusion criteria, aiming for a clearer delineation of the most appropriate patient cohort for TMS in MDD. This entails refining criteria related to treatment history and concomitant medication use to enhance the precision and generalizability of findings.

The uniform application of TMS in the dorsolateral prefrontal cortex or prefrontal cortex across all three studies underscores another noteworthy limitation. The field of TMS is continually evolving, with emerging electrode placements, bilateral approaches, and novel TMS protocols, such as theta burst stimulation, warranting exploration. A more diversified array of lead placements, pulse frequencies, and pulse quantities must be investigated to establish evidence‐based treatment protocols for depression during pregnancy. The largely uniform application of TMS across the three studies included in this review limits the ability to compare and contrast, and subsequently develop recommendations for the most appropriate TMS administration protocol during pregnancy.

Furthermore, the dearth of long‐term studies tracking the neurodevelopmental progress of offspring whose mothers underwent TMS treatment during pregnancy is a conspicuous gap in the literature. To date, only one such study exists (Eryılmaz et al. 2015). This was a study conducted by Eryilmaz et al. in 2014, offering insights into the cognitive and motor development of children born to mothers who received TMS during pregnancy. While the results from this study were promising, the small sample size remains a critical limitation. Given the relatively short timeframe between FDA approval of TMS in 2008 and publishing of this study in 2013, understandably, these children were very young at follow up—with a mean age of 32 months (range 16–64 months). Longer‐term studies, including follow‐up of children into adolescence and beyond, will be crucial to more wholly explore long‐term outcomes of children with mothers treated with TMS during their pregnancy. Thus, larger, more comprehensive longitudinal studies are essential to validate these preliminary findings and comprehensively assess the potential effects of TMS on offspring development.

Despite the constraints posed by small sample sizes in several of the reviewed studies, the findings concerning the use of TMS during pregnancy are encouraging. If further RCTs and longitudinal investigations continue to substantiate the efficacy and safety of TMS in this context, it could represent a significant advancement in the field of perinatal psychiatry. TMS has the potential to serve as a noninvasive, well‐tolerated intervention, offering considerable promise. Presently, TMS is accessible in both inpatient and outpatient settings worldwide. As research in this area progresses, enhanced accessibility and expansion to primary care and obstetric clinics have the potential to revolutionize perinatal mental health care, greatly benefitting pregnant individuals dealing with major depressive disorder.

## 5 Conclusions

Our analysis of the available literature underscores the potential of TMS targeted at the dorsolateral prefrontal cortex as a safe and effective treatment for depression during pregnancy. All three studies encompassed in this review reported significant reductions in depression severity scores, consistent tolerability among the pregnant women, and minimal to negligible adverse neonatal outcomes. However, to enhance our understanding and affirm the safety and efficacy of TMS in managing depression during pregnancy, further research is imperative.

The imperative need for more RCTs and larger, multicenter studies emerges as a central recommendation. These endeavors should aim to encompass a more extensive participant base and investigate varied electrode placements, taking advantage of recent innovations in TMS techniques, such as theta burst stimulation. Comparative RCTs exploring different TMS modalities will contribute to the development of evidence‐based TMS protocols tailored for use during pregnancy. Additionally, larger studies differentiating between participants with treatment‐resistant major depressive disorder, major depressive disorder and MDD with TMS used as an adjunct to pharmacology will allow better elucidation of the most appropriate cohort for treatment with TMS.

Furthermore, while our reviewed studies primarily examined short‐term outcomes in offspring of mothers who underwent TMS during pregnancy, we call for a more extensive exploration of medium to long‐term outcomes in children and adolescents. This longitudinal perspective is critical to comprehensively evaluate the physical and mental health outcomes in offspring whose mothers received TMS during pregnancy.

In summary, the current evidence suggests that TMS holds promise as a valuable therapeutic option for pregnant individuals experiencing depression. To validate and expand upon these preliminary findings, additional high‐quality research is essential, encompassing larger and more diversified study populations, more in‐depth examination of TMS modalities, and longer‐term follow‐up studies of offspring. Ultimately, such investigations have the potential to enhance perinatal mental health care by providing evidence‐based guidelines for the use of TMS during pregnancy (Table A1).

## Author Contributions

**Serena Angeline**: writing–original draft, writing–review and editing, data curation, formal analysis. **Babangida Tiyatiye**: writing–review and editing, methodology. **Wole Akosile**: conceptualization, writing–review and editing, project administration, supervision, methodology.

### Conflicts of Interest

The authors declare no conflict of interest.

### Peer Review

The peer review history for this article is available at https://publons.com/publon/10.1002/brb3.70304.

## Data Availability

No new data were created or analyzed in this study. Data sharing is not applicable to this article.

## 1

**TABLE A1:** Risk of assessment bias using ROBINS, JADDAD tools.

Author	Bias due to confounding	Bias in selection of participants into the study	Bias in classification of interventions	Bias due to diversions from intended interventions	Bias due to missing data	Bias in measurement of outcomes	Bias in selection of the reported result	Overall bias
**Kim et al**. (2011)	**Serious** (no analysis of confounding factors)	**Moderate**	**Low**	**Low**	**Low**	**Serious** (no binding)	**Low** (all data reported)	**Serious**
**Hızlı Sayar et al**. (2014)	**Serious** (no analysis of CF)	**Moderate**	**Low**	**Low**	**Low**	**Serious** (no blinding)	**Low**	**Serious**
**Study**	**Randomized as described**	**Appropriate randomization**		**Described as double blind**	**Appropriate blinding**	**Description of withdrawals/dropouts**		**Overall risk of bias**
**Kim et al**. (2019)	**Yes**	**Yes**		**Yes**	**Yes**	**Yes**		**Low**
